# Complete genome sequence of the novel duck hepatitis B virus strain SCP01 from Sichuan Cherry Valley duck

**DOI:** 10.1186/s40064-016-2988-5

**Published:** 2016-08-17

**Authors:** Qingqing Li, Renyong Jia, Siyang Liu, Mingshu Wang, Dekang Zhu, Shun Chen, Mafeng Liu, Zhongqiong Yin, Bo Jing, Anchun Cheng

**Affiliations:** 1 Avian Disease Research Center, Sichuan Agricultural University, Chengdu, 611130 China; 2 Institute of Preventive Veterinary Medicine, Sichuan Agricultural University, Chengdu, 611130 China; 3 Key Laboratory of Animal Disease and Human Health of Sichuan Province, Chengdu, 611130 China

**Keywords:** Duck hepatitis B virus, Cherry valley duck, Phylogenetic analysis, Complete genome sequence, Chinese and Western isolates

## Abstract

**Background:**

The duck hepatitis B virus (DHBV) strain, designated SCP01, was isolated and identified from a Sichuan Cherry Valley duck in Southwestern China. To determine the origination and evolution of this isolated strain, we carried out complete genome sequencing of this strain.

**Findings:**

Sequencing of the nucleotide sequence of DHBV strain SCP01 revealed a genome size of 3021 bp that contained three open reading frames, designated as C, S, and P, which were consistent with those of other duck hepatitis B viruses nucleotide sequences available in the GenBank of NCBI. Sequence comparisons based on the full-length genomic sequences showed that the DHBV SCP01 strain had the highest similarity (99.64 %) with the sequence of strain DHBV-XY, but had a lower similarity (90.04 %) with the sequence of strain DHBV CH5 isolated from Southwestern China. Phylogenetic analysis revealed that the DHBV-XY and DHBV SCP01 formed a branch that was clearly distinct from the other strains.

**Conclusion:**

This study show that the DHBV SCP01 strain from Sichuan belonged to “Western” isolates, while the DHBV CH5 from Sichuan belonged to “Chinese” isolates. These data will promote further research into the evolutionary biology, epidemiology and pathobiology of hepadnavirus infections. In addition, continuing duck hepatitis B virus surveillance in poultry is critical to understand the patterns of DHBV infection, and to find further animal infection models to study HBV infection.

## Background

Duck hepatitis B virus (DHBV), first discovered in Peking ducks in 1980 (Mason et al. 1980), and subsequently reported in Germany and other countries throughout the world (Mattes et al. 1990; Triyatni et al. 2001; Mangisa et al. 2004; Liu et al. 2014), is a member of the genus *Avihepadnavirus*, family *Hepadnaviridae*. The genome of DHBV, a complete minus and incomplete plus strand, is circular and approximately 3.0 kb in length (Cova et al. 2011). The genomic DNA is maintained in a circular conformation by a short cohesive overlap between the two DNA strands (Molnar-Kimber et al. 1984). DHBV is similar to hepatitis B virus (HBV) in terms of genetic organization and virus replication, and causes species-specific transient (acute) or persistent infection (Jilbert and Kotlarski 2000). There are approximately 240 million people worldwide that are chronically infected with HBV (Lavanchy and Kane 2016). Chronic HBV infection results in liver disease, including inflammation, fibrosis, cirrhosis and hepatocellular carcinoma (HCC) (Feng et al. 2010). DHBV does not lead to severe clinical disease in ducks or a drop in productivity, but serves as an animal infection model of human HBV, and has been used widely for comparative studies.

## Methods

### Virus isolation

Positive serum screened by PCR using the primers P1 and P2 (Table 1) was collected and filtered through a 0.22 µm filter. The 9-day-old duck embryonated eggs were inoculated with the filtered suspension (100 μL/embryo) into the allantoic cavity and then cultured in a 37 °C incubator and checked daily. The allantoic fluid was harvested at 4 days after inoculation and then for another round of inoculation.

**Table 1:** Primers for PCR amplification

Name	Sequence (5′–3′)
P1	ATGTCTGGTACCTTCGGGGGA
P2	CTAACTCTTGTAAAAAAGAGC
P3	AATTACACCCCTCTCCTTCGGAG
P4	GTAATTCTTAAGTTCCACATAGCC
P5	CACCCCTCTCTCGAAAGCAATA
P6	GATAGTCAGGTTGAAAGCTCAC

### Nucleotide sequencing of the complete genome

**Fig. 1:**
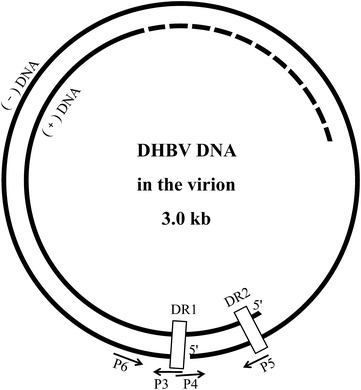
Schematic representation and primers used for amplification of the whole DHBV genomes. The whole DHBV genomes are *open circular* DHBV virion DNA, which contain a complete minus and incomplete plus strand. The genomic DNA is maintained in a circular conformation by a short cohesive overlap between the two DNA strands, the cohesive overlap region is located between DR1 and DR2. The triple-strand region contains the terminal redundany of the minus strand, regions of the viral genome containing DR1 and DR2 correspond to the sites of initiation of synthesis of the viral minus and plus strands. Primer P3 and P4 is used to amplify the complete genome sequence of DHBV in region of DR1, primer P5 and P6 is designed to amplify the sequence including the region of primer P3 and P4 for ensuring the full gene sequence correctly. *DR* direct repeat

Viral DNA was extracted from 100 µL of allantoic fluid using the TIANamp Virus DNA/RNA Kit (TIANGEN BIOTECH, Beijing, China) according to the manufacturer’s instructions. Afterwards, based on the multiple alignments of the complete genome of DHBV available in GenBank, two pairs of primers were designed using Primer Premier 5 software (Table 1; Fig. 1) (Günther et al. 1995). Primers P3 and P4 were designed to amplify the complete genome sequence of DHBV, the PCR amplification was performed in a 50 μL mixture containing 0.5 μL LAmp™ DNA polymerase (5 U/μL), 1 μL dNTP Mix (10 Mm each), 5 μL 10 × LAmp™ buffer (with 20 mM MgCl_2_), 1 μL viral DNA template, 2 μL each of primers P3 and P4 (10 μM), and 38.5 μL ddH_2_O. The amplification procedure consisted of denaturation at 94 °C for 5 min followed by 32 cycles of denaturation at 94 °C for 30 s, annealing at 57 °C for 30 s, extension at 72 °C for 3 min 10 s, and then a final extension at 72 °C for 7 min. Primers P5 and P6 were used to amplify the region including the sequences of P3 and P4, which PCR conditions were similar to the above amplification. The amplified products were purified using the Universal DNA Purification Kit and cloned into the pGM-T vector (TIANGEN BIOTECH), and then sequenced using classical dideoxy Sanger sequencing (TSINGKE, Chengdu, China). The sequences were assembled using the Chromas software package (http://www.Technelysium.Com.au/chromas.html) to produce the final genome sequence.

### Genetic characterization

To exhibit the genome features of the SCP01 strain, sequence analysis was conducted using the DNAMAN program. The open reading frames (ORFs) were identified according to the online tool ORF Finder (http://www.ncbi.nlm.nih.gov/gorf/gorf.html) in National Center for Biotechnology Information (NCBI).

### Phylogenetic analysis

Phylogenetic analyses were performed using the maximum likelihood method with the use of Mega5.1 software (Tamura et al. 2011). Initial trees for the heuristic search were obtained by applying the neighbor-joining method to a matrix of pairwise distances estimated using the maximum composite likelihood approach. The bootstrap consensus trees inferred from 1000 replicates and branches corresponding to partitions reproduced in less than 70 % bootstrap replicates were collapsed.

## Results and discussion

In June 2014, DHBV strain SCP01 was isolated from ducklings in a commercial Cherry Valley duck breeding company in Sichuan Province, Southwestern China. Subsequently, the nucleotide sequence of DHBV strain SCP01 was amplified by PCR using the primers P3 and P4, primers P5 and P6. Then the amplified products were purified and cloned into the pGM-T vector and then sequenced using classical dideoxy Sanger sequencing. Sequencing revealed that the PCR products through primers P3 and P4, primers P5 and P6 corresponded to the predicted length of 3027 and 526 bp in size, respectively, then the sequences were assembled using the Chromas software package to produce the final genome sequence of 3021 bp in length. The isolated virus was identified as DHBV and named SCP01.

Sequence analysis revealed that the genome of DHBV SCP01 (GenBank accession number KM676220) was a double-strand circular DNA and had a size of 3021 bp, with a G + C content of 43.03 %. In addition, the coding region of DHBV SCP01 had three ORFs, designated ORF C, S, and P, which were predicted in the genome by comparison with the proposed structures of other duck hepatitis B viruses nucleotide sequences available in the GenBank of NCBI and identified by the following major criteria: an ATG start codon, a minimum length of 60 bp, and less than 60 % overlap with adjacent ORFs, using the online ORF Finder in NCBI. ORFs S, C and P were predicted to encode the viral surface (preS/S protein: 36.2 kDa and S protein: 18.2 kDa), the core protein (preC/C protein: 35 kDa and C protein: 30.3 kDa), and the polymerase protein (P protein: 89.6 kDa), based on sequence similarities and the presence of conserved domains.

The DHBV SCP01 sequence was aligned with 13 reference strains of DHBV and a Snow goose hepatitis B virus (SGHBV, GenBank accession number AF110998) sequence obtained from GenBank database. The percent nucleotide identity and amino acid identity of DHBV strain SCP01 with those of other avian hepadnaviruses are summarized in Table 2. The DHBV SCP01 genome is the same size as the Western isolates AY250902.1 (Mangisa et al. 2004), AY250904.1 (Mangisa et al. 2004), X12798.1 (Mattes et al. 1990), DQ195079.1 (Sprengel et al. 1985), HQ214130.1, and HQ132730.1. The DHBV SCP01 isolate was 3 nt shorter than the AY494851.1 (Guo et al. 2005), DQ276978.1 and AF110998.1 (SGHBV) (Chang et al. 1999); and 6 nt shorter than the China isolates HM043822.1, AY392760.1, JX469897.1, and M32991.1 (Masako et al. 1989). The differences in length were the result of differences at nt positions 1239–1241 and 1277–1279 of the 3027 nt genome.

**Table 2:** Similarities analysis with the 15 isolates of avian hepadnavirus

Number	GenBank	Complete genome^a^	Polymerase protein^b^	Genomic length (nt)	Avain species	Location
1	AY250902.1	99.47	99.49	3021	Pekin duck	South Africa
2	AY250904.1	99.37	99.24	3021	Pekin duck	South Africa
3	X12798.1	98.58	98.09	3021	Duck	Germany
4	DQ195079.1	94.24	91.09	3021	Pekin duck	Germany
5	AY494851.1	91.39	88.04	3024	Puna teal	USA
6	HQ214130.1	**99.64**	**99.62**	3021	Cherry valley duck	Henan
7	HQ132730.1	94.47	91.60	3021	Duck	Guangdong
8	DQ276978.1	90.40	91.48	3024	Tadorna tadorna	Hubei
9	HM043822.1	90.07	91.35	3027	Cherry valley duck	Henan
10	EU429325.1	90.04	87.91	3006	Anas platyrhynchos	Sichuan
11	AY392760.1	90.93	86.64	3027	Brown duck	Guangdong
12	JX469897.1	90.07	86.51	3027	Mallard	Guilin
13	M32991.1	*89.47*	*84.61*	3027	White Shanghai duck	Shanghai
14	AF110998.1	89.14	85.37	3024	Anser caerulescens	Germany
15	KM676220.1			3021	Cherry valley duck	Sichuan

Boldface indicates the highest, and italic, the lower

^a^Percent nucleotide identity

^b^Percent amino acid identity

**Fig. 2:**
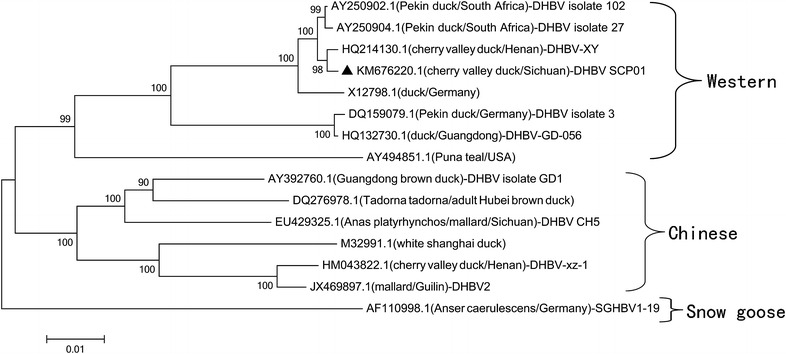
Phylogenetic analysis of the complete genomic sequences of DHBV SCP01. The host and geographic origin are shown in *brackets*. The phylogenetic tree was constructed using the neighbor-joining method implemented in MEGA5 software, with 1000 bootstrap replicates to assign confidence to groupings. All the accession numbers correspond to GenBank Submissions of different strains of the avian hepadnaviruses. The isolate DHBV SCP01 is marked with a filled triangle. The complete nucleotide sequence of SGHBV was used as the phylogenetic outgroup. The *scale bar* indicates the number of substitutions per residue

Sequence alignment between the reference strains and the DHBV SCP01 was performed using the Mega 5.1 software, and the neighbor-joining method (Clustal W) was applied. The corresponding phylogenetic tree was constructed using the sequence data of SGHBV, a member of the *Avihepadnavirus*, as the outgroup. The phylogenetic relationships are presented in Fig. 2. DHBV-XY (GenBank accession number HQ214130.1; origin, Xinyang, China) and DHBV SCP01 formed a branch that was clearly distinct from the other strains. We found that the DHBV SCP01 strain had the highest similarity (99.64 %) with the sequence of strain DHBV-XY, but had a lower similarity (90.04 %) with the sequence of strain DHBV CH5 (GenBank accession number EU429325.1; origin, Sichuan, China) isolated from Southwestern China. Comparing the DHBV SCP01 strain with other isolated DHBV strains, the ORF S, C, and P similarities were approximately 88.35–99.90, 86.60–99.78, and 89.62–99.62 %, respectively, and the complete genome sequence similarity was approximately 89.47–99.64 %. This report will aid our understanding of the epidemiology and molecular characteristics of DHBV from Cherry Valley ducks in Southwestern China.

The phylogenetic analysis of the entire nucleotide sequence indicated that the DHBV strain SCP01 was clustered with the strains from Western countries, and it was more closely related to DHBV-XY from central China, than to strains isolated from other areas of China, France, Canada, India, South Africa, Australia, Germany, and the United States. The DHBV strain SCP01 and DHBV-XY belong to “Western” isolates, while others (especially DHBV CH5) belong to “Chinese” isolates. This might be because of the introduction of exotic ducks between countries in recent years during rapid economic development. Phylogenetic analysis of the individual ORFs did not alter the position of the DHBV SCP01 isolate in the trees. Upon translation of ORF P, the DHBV SCP01 isolates were found to share signature amino acids with the Western isolates, as opposed to those of the Chinese isolates (Table 3). The translated sequences of the DHBV SCP01 isolates had a few amino acid changes when compared with the sequences of Western isolates, especially between the DHBV-XY and the DHBV strain SCP01, which were mostly in the polymerase protein (three amino acid changes).

**Table 3:** Comparison of amino acid residues of DHBV SCP01 isolates with those of other avain hepadnaviruses

ORF region	Signature amino acid residue^a^	HQ214130 DHBV-XY	KM676220 DHBV SCP01	Western isolates	Chinese isolates	EU429325.1DHBV CH5	Exceptions^b^
Polymerase Spacer	202	H	H	H	Y	Y	
	208	Q	H	H	Q	Q	
	210	Y	Y	Y	P	P	
	223	T	T	T	E	E	
	231	V	V	V	A	A	
	237	K	K	K	P	P	
	244	F	F	F	C	S	
	272	S	S	S	T^b^	S	X58569(P)
	345	S	S	S	Q	Q	
	356	V	V	V	E	E	
	360	G	G	G	R	R	
	566	N	N	N	K^b^	K	X58568/9(R)
	635	S	S	S	A	A	
	694	V	V	V	I	I	
	700	S	S	S	V	V	
	769	P	P	P	T	T	

^a^Numbering of amino acid residues is according to 3021 base pair complete genome

^b^Exceptions: the alternative amino acid residue is shown in the square brackets

## Conclusion

This study show that the DHBV SCP01 strain from Sichuan belonged to “Western” isolates, while the DHBV CH5 from Sichuan belonged to “Chinese” isolates. The complete molecular characterization of the DHBV SCP01 strain will contribute to further studies on molecular epidemiology and enable the development of better measures to control DHBV. To date, there are no effective, preventive vaccines against DHBV in poultry, and the nature of circulating DHBV and HBV have remained largely elusive in China. Our purpose in submitting this report is our hope that these data will promote investigations by others in the virology community into the evolutionary biology, epidemiology and pathobiology of hepadnavirus infections. In addition, continuing duck hepatitis B virus surveillance in poultry is critical to understanding DHBV infection, and to find further animal infection models to study HBV infection.

## Abbreviations

DHBV: duck hepatitis B virus
HBV: hepatitis B virus
SGHBV: snow goose hepatitis B virus
HCC: hepatocellular carcinoma
NCBI: National Center for Biotechnology Information
ORFs: open reading frames
DR: direct repeat
